# Acute Macular Neuroretinopathy Following COVID-19 Infection: A Case Report

**DOI:** 10.7759/cureus.78182

**Published:** 2025-01-29

**Authors:** Ryan Zubricky, William Ensor

**Affiliations:** 1 Ophthalmology, Geisinger Health System, Danville, USA

**Keywords:** acute macular neuroretinopathy, coronavirus, covid-19, ophthalmology, retina

## Abstract

A 24-year-old female presented with a new scotoma in her left eye one week after testing positive for COVID-19. Fundoscopy revealed an inferonasal perifoveal retinal pigment epithelial (RPE) lesion in the left eye. Optical coherence tomography (OCT) displayed an area of outer retinal defect involving the photoreceptor layer with ellipsoid zone disruption. OCT angiography (OCTA) showed decreased reflectivity in the deep capillary plexus of the affected area. The patient was diagnosed with acute macular neuroretinopathy (AMN) and monitored without treatment. At a one-month follow-up, minimal improvement in the scotoma was noted, with OCT showing mild improvement. At four months, the scotoma remained stable, with a persistent outer retinal defect on OCT.

Despite the persistent scotoma and outer retinal defect, the patient maintained good central vision. Clinicians should be aware of potential ocular complications of COVID-19, including AMN, and monitor patients accordingly. Currently, there is no evidence-based treatment for AMN, and long-term follow-up studies are needed to better understand the prognosis and potential treatments for this condition.

## Introduction

Acute macular neuroretinopathy (AMN) is a rare condition characterized by sudden vision loss in the central visual field, presenting as one or more scotomas, with findings of neuroretinal dysfunction and atrophy in one or both eyes. The exact pathogenesis of AMN remains unclear, though it is thought to result from ischemia of the deep capillary plexus [1]. Diagnosis is made through clinical correlation of symptoms and characteristic macular lesions with outer retinal loss on OCT [1]. The reported prevalence is less than 1 patient per million, though this is likely an underestimate due to the rarity of the disease [2]. Recent reports have suggested an association between AMN and COVID-19 infection, with an increased incidence over the past few years linked to both COVID-19 infection and vaccination, though no population studies have been conducted [2-7]. AMN most commonly affects young women, a trend that continues with presumed COVID-associated AMN [6]. Visual outcomes are mixed, with some patients experiencing a persistent scotoma that does not resolve [8].

## Case presentation

**Figure 1:**
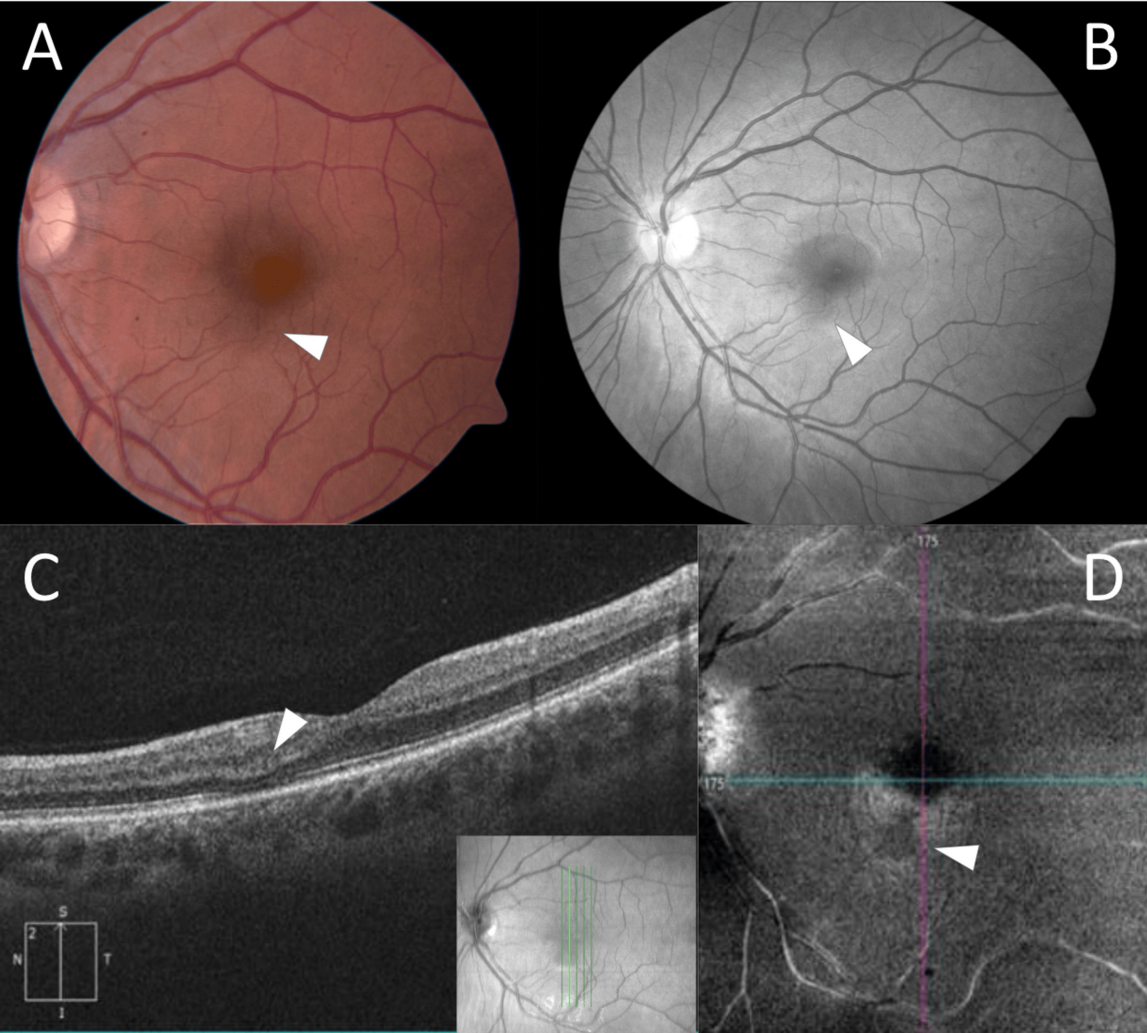
Multimodal Fundus Imaging A. Fundus photograph displaying subtle pigmented round lesion inferior to fovea. B. Red-free fundus photograph displaying inferior lesion. C. OCT displaying focal loss of ellipsoid zone in area of lesion with depression of overlying outer nuclear and plexiform layers. D. OCTA showing decreased reflectivity in deep retinal layers corresponding with lesion. OCT: optical coherence tomography, OCTA: optical coherence tomography angiography.

A 24-year-old female presented with a new paracentral scotoma in her left eye for the past three days following mild flu-like symptoms approximately one week prior and testing positive for COVID-19 via rapid antigen test. She denied other ocular symptoms, including new floaters, changes in visual acuity, color vision, contrast sensitivity, or night vision. She was not taking any medications and had no known family history of ocular conditions. She had not received any COVID-19 vaccinations. Ocular examination revealed visual acuity of 20/20 in the right eye and 20/25 in the left. Refractive error by manifest refraction was -4.25 + 1.00 x 087 in the right eye and -4.50 + 1.50 x 071 in the left. Intraocular pressures and pupils were normal, with full confrontational visual fields. A slit lamp exam showed no abnormalities in the anterior segment. Fundoscopy revealed an inferonasal perifoveal retinal pigment epithelium (RPE) lesion in the left eye. Optical coherence tomography (OCT) displayed an area of outer retinal defect involving the photoreceptor layer with disruption of the ellipsoid zone, consistent with the lesion and the described scotoma. OCT angiography (OCTA) showed decreased reflectivity in the deep capillary plexus corresponding to the area of the lesion (Figure 1).

A diagnosis of AMN was made, and the patient was counseled to monitor her symptoms without any treatment. At a one-month follow-up, the patient reported minimal subjective improvement in her scotoma. Visual acuity remained stable, with repeat examination and OCT showing mild improvement in the defect. At a four-month follow-up, the patient noted no changes in the scotoma, and OCT demonstrated a stable outer retinal defect.

## Discussion

The findings in this case support the current literature on the association between COVID-19 and AMN. Since the beginning of the COVID-19 pandemic, there has been a notable increase in reported AMN cases, with several case series documenting this association [2,6]. While AMN has several known environmental risk factors, including flu-like illness, oral contraceptive use, exposure to epinephrine/ephedrine, trauma, and hypovolemia [1], the connection with COVID-19 appears particularly significant due to the virus’s pathophysiology.

The pathogenesis of AMN in COVID-19 infection likely involves multiple mechanisms. The primary theory suggests ischemia of the deep capillary plexus leading to hypoxia of the middle and outer retina [1], supported in this case by the hyporeflective lesion in the deep retinal layers on OCTA. COVID-19 infection creates a particularly conducive environment for this process through several mechanisms. First, the virus triggers a hypercoagulable state characterized by elevated D-dimer levels and increased inflammatory markers [6]. Second, direct endothelial cell damage and dysfunction lead to microvascular complications [9]. A recent case series suggested that this combination of increased thrombotic formation and cell-mediated vasoconstriction could drive the ischemic insult [6].

The imaging findings in our case align with typical AMN features but also demonstrate characteristics commonly seen in COVID-associated cases. The OCT findings of outer retinal defect and ellipsoid zone disruption are classical for AMN, while the OCTA demonstration of decreased deep capillary plexus flow is particularly notable. Although our patient presented with unilateral disease, bilateral involvement appears more common in COVID-associated AMN, reported in up to 70% of cases in recent series, fitting with systemic hypercoagulability [6]. This difference in presentation may reflect varying degrees of systemic involvement or differences in the timing of diagnosis.

The prognosis of COVID-associated AMN appears similar to traditional cases, though some studies suggest a higher rate of persistent scotomas [8]. In our patient, the scotoma remained stable with minimal improvement, consistent with recent reports showing that 60% of COVID-associated AMN cases exhibit persistent visual symptoms [8]. The natural history of these lesions typically shows stability or mild improvement over time, though complete resolution is rare.

Regarding treatment, there remains no evidence-based standard of care for AMN. While some case reports have documented improvement with oral steroids [7,10], the role of anti-inflammatory treatment remains controversial. The theoretical basis for steroid use is centered on reducing inflammation, particularly relevant in COVID-associated cases due to the virus's inflammatory effects. However, no large comparative studies have demonstrated a clear benefit. Some authors suggest that early intervention with steroids might be more beneficial in COVID-associated cases due to the significant inflammatory component [7], though this requires further investigation. Additionally, there are no current recommendations for systemic workup or neuroimaging. AMN remains a rare disease with no known systemic associations that would necessitate testing based on the presence of AMN alone.

Despite the persistent scotoma and outer retinal defect in our patient, her central vision remained intact, with a good overall visual prognosis. This outcome aligns with the typical course of AMN, where central visual acuity often remains preserved despite persistent paracentral scotomas.

## Conclusions

This case highlights the presumed association between COVID-19 infection and AMN and underscores the need for further investigation into the underlying mechanisms. Despite its high prevalence, COVID-19 is thought to be a causative factor in patients presenting with AMN, which has seen an increase in prevalence since the outbreak of COVID-19 in 2020. Clinicians should be aware of the potential ocular complications of COVID-19, including AMN, and monitor patients with new onset visual symptoms accordingly. This case emphasizes the importance of ophthalmologic examination in patients with new visual symptoms during or after COVID-19 infection. Currently, there is no evidence-based treatment for AMN. Long-term follow-up and comparative studies are needed to further elucidate the role of steroid treatment and to better understand the prognosis of AMN.
